# Use of Community-Based Participatory Research Partnerships to Reduce COVID-19 Disparities Among Marshallese Pacific Islander and Latino Communities – Benton and Washington Counties, Arkansas, April–December 2020

**DOI:** 10.5888/pcd18.210124

**Published:** 2021-10-07

**Authors:** Pearl A. McElfish, Brett Rowland, Austin Porter, Holly C. Felix, James P. Selig, Judd Semingson, Don E. Willis, Michelle Smith, Sheldon Riklon, Eldon Alik, Alan Padilla-Ramos, Erika Y. Jasso, Namvar Zohoori

**Affiliations:** 1 College of Medicine, University of Arkansas for Medical Sciences Northwest, Fayetteville, Arkansas; 2 Office of Community Health and Research, University of Arkansas for Medical Sciences Northwest, Fayetteville, Arkansas; 3 Arkansas Department of Health, Little Rock, Arkansas; 4 Fay W. Boozman College of Public Health, University of Arkansas for Medical Sciences, Little Rock, Arkansas; 5 Community Clinic, Springdale, Arkansas; 6 Republic of the Marshall Islands Consulate, Springdale, Arkansas

## Abstract

Marshallese and Latino communities in Benton and Washington counties, Arkansas, were disproportionately affected by COVID-19. We evaluated the effectiveness of a comprehensive community-based intervention to reduce COVID-19 disparities in these communities. We examined all laboratory-confirmed COVID-19 cases in the 2 counties reported from April 6, 2020, through December 28, 2020. A 2-sample serial *t* test for rate change was used to evaluate changes in case rates before and after implementation of the intervention. After implementation, the proportions of cases among Marshallese and Latino residents declined substantially and began to align more closely with the proportions of these 2 populations in the 2 counties. Infection rates remained lower throughout the evaluation period, and weekly incidence also approximated Marshallese and Latino population proportions. Leveraging community partnerships and tailoring activities to specific communities can successfully reduce disparities in incidence among populations at high-risk for COVID-19 .

**Summary**

**What is already known on this topic?**

As of June 13, 2020, Marshallese and Latino residents of 2 Arkansas counties made up approximately 19% of the total state population but accounted for 64% of COVID-19 cases according to the Centers for Disease Control and Prevention (CDC).

**What is added by this report?**

By using community partnerships and CDC recommendations, the Comprehensive Intervention to Reduce COVID-19 Disparities in Marshallese and Latinx Communities was developed and implemented, resulting in reduced incidence of COVID-19 that was sustained.

**What are the implications for public health practice?**

The findings highlight the importance of community partnerships in addressing health disparities among people at risk for COVID-19. Community partnerships will continue to be leveraged to promote COVID-19 vaccine distribution and uptake.

## Introduction

COVID-19 infections, hospitalizations, and deaths have disproportionately affected minority communities in the US (1). Many minority communities have a higher prevalence of chronic conditions such as type 2 diabetes (2), which has been shown to increase hospitalization and mortality rates for COVID-19 (3). Marshallese Pacific Islanders and Latino community members in Benton and Washington counties, Arkansas, have experienced significant disparities in COVID-19 infections, hospitalizations, and deaths. (4,5). These 2 communities face similar socioeconomic challenges, including low-wage employment, lack of health insurance, and unstable and dense housing, all of which elevate their risk of contracting COVID-19 (5). Community-level investigations conducted by the Centers for Disease Control and Prevention (CDC) found that 19% of all COVID-19 infections in Benton and Washington counties as of June 13, 2020, were among Marshallese residents, and 45% were among Latino residents (4,5). The Marshallese community accounts for only 2.4% of the 2-county population, and the Latino community accounts for 17%.

Hospitalizations and deaths were also much higher among Marshallese residents. Five percent of total COVID-19 cases in the 2 counties resulted in hospitalization, compared with 9% for Marshallese residents alone (5). Marshallese residents accounted for 38% of all COVID-19 deaths in the 2 counties (83.3 deaths per 100,000 residents) (5). These disparities may be attributed to the high prevalence of chronic diseases such as obesity, type 2 diabetes, and hypertension among the Marshallese population. A local needs assessment conducted among Marshallese adults (N = 401) found that 61.7% were obese, 38.4% had HbA_1c_ (hemoglobin A_1c_) levels indicative of type 2 diabetes, and 41.2% had blood pressures indicative of hypertension (6).

## Purpose and Objectives

CDC’s report highlighted confusion among Marshallese and Latino community members about prevention behaviors, testing, isolation and quarantine, and available services and noted these residents were often unaware of or unable to access support services available in the local community (4,5). The report concluded, “reducing COVID-19 disparities requires strengthening the coordination of public health, health care, and community stakeholders to provide tailored health education, community-based prevention activities, case management, care navigation, and service linkage” (4).

On the basis of CDC’s findings, we leveraged existing community and health care system partnerships to develop and deploy the Comprehensive Intervention to Reduce COVID-19 Disparities in Marshallese and Latinx Communities. This article describes the evaluation of the effectiveness of this intervention by examining changes in COVID-19 infections following the intervention’s implementation among these communities.

## Intervention Approach

The University of Arkansas for Medical Sciences (UAMS) has worked with the Marshallese and Latino communities since 2013 to address chronic disease disparities and social determinants of health by using a community-based participatory research (CBPR) approach (7). CBPR can build trust between academic health care providers, researchers, and the community by engaging the community and honoring their unique contributions at all stages of an intervention (8). By using a CBPR approach, our partners’ cultural knowledge and expertise were integrated into the intervention, ensuring cultural appropriateness.

Since March 2020, our community–academic partnership has met weekly, often with daily communication among partners. Local partners included nonprofit organizations serving the Marshallese and Latino communities, community-based nonprofit organizations, local hospital systems, a federally qualified health center, the Veterans Administration, the Arkansas Department of Health (ADH), and the Republic of the Marshall Islands consulate. Our partnerships were leveraged to develop and implement the Comprehensive Intervention to Reduce COVID-19 Disparities in Marshallese and Latinx Communities in Benton and Washington counties. The comprehensive intervention consisted of 4 components: 1) health education, 2) testing, 3) contact tracing, and 4) care navigation (case management) for supported quarantine. UAMS served as a single coordination center for the intervention components. Full details of the comprehensive intervention have been published elsewhere (9,10).

**Health education and prevention.** The intervention included a culturally appropriate communications plan designed to reach Marshallese and Latino community members. The culturally tailored messages focused on COVID-19 prevention, testing, quarantine, isolation, and follow-up care. Communications were provided in English, Marshallese, and Spanish, and all communications featured local Marshallese and Latino community leaders. We used both formal and informal distribution methods, including Facebook posts, Facebook live videos, YouTube videos, printed materials, radio, billboards, and television (https://nwacouncil.org/covid-resources/).

**Testing**. The intervention included multiple testing strategies, including community-based drive-through testing sites, clinic-based testing, and home-based serial testing. All tests were provided free of charge, and all testing events were staffed with at least one bilingual medical interpreter each for English/Marshallese and English/Spanish to ensure services were culturally and linguistically appropriate for the target communities.

**Trilingual contact tracing center**. The intervention provided contact tracing in 3 languages at a dedicated center with a bilingual staff (ie, English, Marshallese, and Spanish). A COVID-19 diagnosis is a mandatory reportable event and must be reported to ADH (11). After cases were reported, those whose preferred language was Marshallese or Spanish were referred to the contact tracing center. A bilingual case worker contacted all people diagnosed with COVID-19 and all contacts.

**Care navigation (case management) for supported quarantine**. Marshallese and Latino communities face many socioeconomic challenges, and these are often exacerbated by a COVID-19 diagnosis, which requires time off from work and additional personal and household expenses. A team of social workers, nurses, and community health workers helped people in quarantine to access essential items, such as food and medications, and coordinated with worksites and community social services agencies. Implementation of the intervention’s health education and free drive-through testing components began July 22, 2020, immediately after the release of the CDC investigation report (4). On August 13, 2020, the intervention received state funding through the CARES Act (12), and on September 1, 2020, the serial testing, contact tracing center, and care navigation for supported quarantine components were fully operational and integrated.

## Evaluation Methods

The first confirmed COVID-19 infection in the Latino community was reported in the week of April 6, 2020, and the first in the Marshallese community, in the week of April 13, 2020. To evaluate the effects of the comprehensive intervention, we examined all laboratory-confirmed COVID-19 cases reported to ADH among Marshallese, Latino, and all other races and ethnicities in the 2 counties from April 6, 2020, through December 28, 2020. Rates of COVID-19 cases per 1,000 residents were calculated by using as denominators previous population estimates in the CDC field report (5), which were derived from 2018 US Census Bureau estimates. Population estimates used for calculating case rates among Latino residents (n = 86,581) and residents of all other races and ethnicities (n = 415,276) are consistent with the CDC report. To evaluate change in case rates before implementation (April 6, 2020–July 13, 2020) versus after (July 20, 2020–December 28, 2020), we used the 2-sample serial *t* test for rate change from Tang and Landes to test for differences in linear trend between the 2 series for these periods (13). We used Fuller’s method to estimate serial correlation (14).

## Results

**Figure 1:**
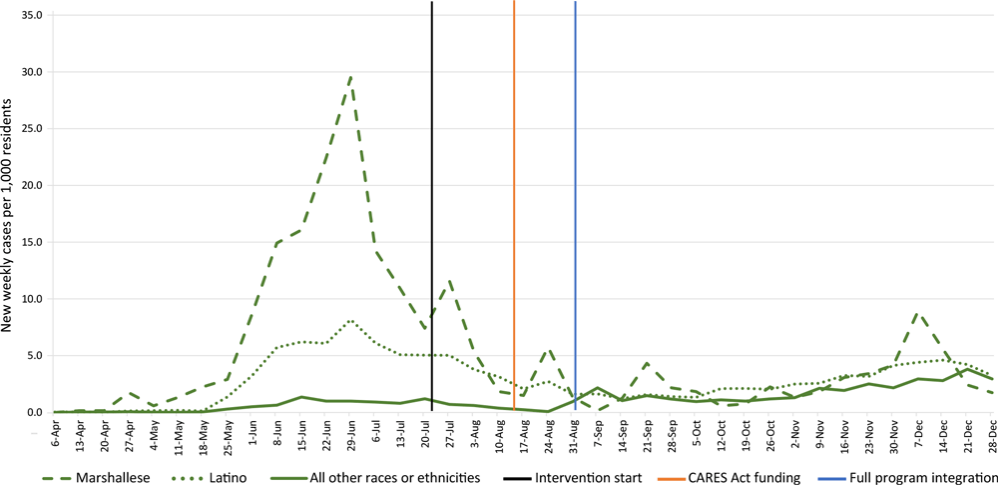
New weekly COVID-19 cases per 1,000 residents, by race or ethnicity, Benton and Washington counties, Arkansas, April–December 2020. The intervention was implemented on July 22, 2020, received state funding through the CARES Act (12) on August 13, 2020, and by September 1, 2020, the serial testing, contact tracing center, and care navigation for supported quarantine components were fully operational and integrated.

New weekly case rates among Marshallese and Latino residents peaked during the week of June 29, 2020, with 29.5 cases per 1,000 (354 new cases) among Marshallese residents and 8.2 cases per 1,000 (708 new cases) among Latino residents (Figure 1). The new weekly case rate among all other racial or ethnic groups for the week of June 29, 2020, was 1.0 case per 1,000 (416 new cases). By December 28, 2020, the new weekly case rate had decreased to 1.8 cases per 1,000 (21 new cases) among Marshallese residents and 3.3 cases per 1,000 (282 new cases) among Latino residents. The new weekly case rate among all other racial or ethnic groups rose to 3.0 cases per 1,000 (1,230 new cases). Significant differences were found in rates of change in cases per 1,000 for both Marshallese (β_pre_ = 1.68; β_post_ = −0.07; *t*(11.24) = −3.76; *P* = .003) and Latino (β_pre_ = 0.60; β_post_ = 0.02;* t*(4.81) = −2.72; *P* = .04), but no significant difference was found for all others (β_pre_ = 0.09; β_post_ = 0.11; *t*(13.34) = 0.49; *P* = .64).

**Table:** 

Date	Marshallese	Latino	All Other Races or Ethnicities
April 6	0.0	0.0	0.0
April 13	0.2	0.0	0.0
April 20	0.2	0.0	0.0
April 27	1.8	0.1	0.1
May 4	0.6	0.2	0.0
May 11	1.3	0.2	0.0
May 18	2.3	0.1	0.1
May 25	2.9	1.4	0.3
June 1	8.7	3.2	0.5
June 8	14.9	5.7	0.6
June 15	16.1	6.2	1.4
June 22	22.4	6.1	1.0
June 29	29.5	8.2	1.0
July 6	14.3	6.1	0.9
July 13	10.9	5.1	0.8
July 20	7.4	5.0	1.2
July 27	11.6	5.0	0.7
August 3	5.3	3.8	0.6
August 10	1.8	3.2	0.4
August 17	1.5	2.1	0.2
August 24	5.8	2.7	0.1
August 31	1.3	1.6	1.0
September 7	0.2	1.6	2.2
September 14	1.3	1.2	1.0
September 21	4.3	1.6	1.5
September 28	2.2	1.4	1.2
October 5	1.8	1.3	1.0
October 12	0.6	2.1	1.1
October 19	0.8	2.1	1.0
October 26	2.3	2.0	1.2
November 2	1.3	2.5	1.3
November 9	1.8	2.6	2.1
November 16	3.1	3.3	1.9
November 23	3.4	3.2	2.5
November 30	4.2	4.1	2.2
December 7	8.9	4.4	3.0
December 14	5.6	4.6	2.8
December 21	2.4	4.2	3.8
December 28	1.8	3.3	3.0

**Figure 2:**
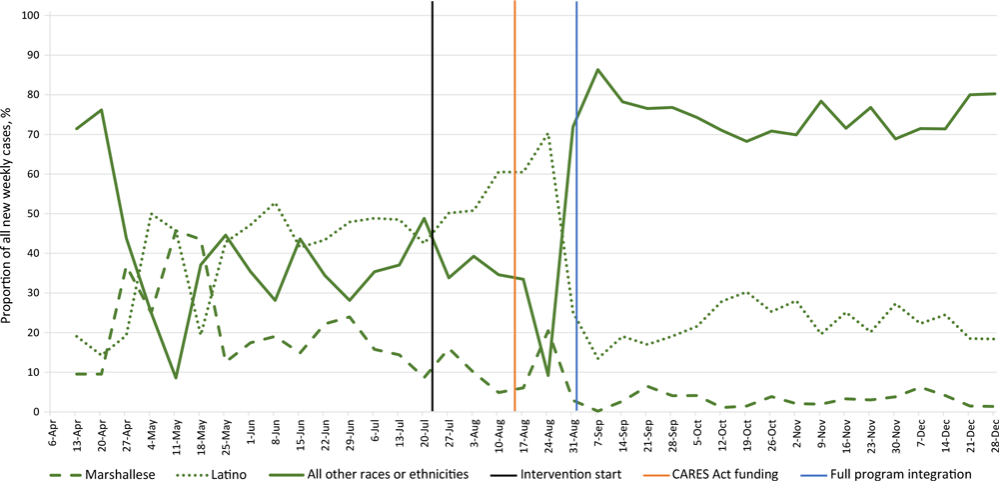
Percentages of new weekly COVID-19 cases among Marshallese and Latino residents, by race or ethnicity, Benton and Washington counties, Arkansas, April–December 2020. The intervention was implemented on July 22, 2020, received state funding through the CARES Act (12) on August 13, 2020, and by September 1, 2020, the serial testing, contact tracing center, and care navigation for supported quarantine components were fully operational and integrated.

The proportions of new weekly cases among Marshallese and Latino residents increased steadily from April to July and continued to increase into August among the Latino population. The highest proportion of new weekly cases among Marshallese residents was 45.7% for the week of May 11, 2020. The highest proportion of new weekly cases among Latino residents was 70.3% for the week of August 24, 2020 (Figure 2). After implementation of the comprehensive intervention, the proportions of new weekly cases among Marshallese and Latino residents declined and began to align more closely with the proportions of Marshallese and Latino community members in the 2 counties.

**Table:** 

Date	Marshallese	Latino	All Other Races or Ethnicities
April 6	0	0	0
April 13	9.5	19.0	71.4
April 20	9.5	14.3	76.2
April 27	36.8	19.3	43.9
May 4	25.0	50.0	25.0
May 11	45.7	45.7	8.6
May 18	43.5	19.4	37.1
May 25	12.6	42.8	44.6
June 1	17.4	47.1	35.4
June 8	19.1	52.8	28.1
June 15	14.9	41.5	43.6
June 22	22.2	43.4	34.4
June 29	24.0	47.9	28.1
July 6	15.8	48.8	35.4
July 13	14.4	48.5	37.1
July 20	8.7	42.5	48.8
July 27	16.0	50.2	33.8
August 3	9.9	50.8	39.3
August 10	4.9	60.5	34.6
August 17	6.1	60.5	33.4
August 24	20.5	70.3	9.2
August 31	2.9	25.2	71.9
September 7	0.2	13.5	86.3
September 14	2.7	19.1	78.2
September 21	6.5	17.0	76.5
September 28	4.1	19.1	76.8
October 5	4.1	21.6	74.3
October 12	1.1	27.9	71.0
October 19	1.5	30.2	68.3
October 26	3.9	25.3	70.9
November 2	2.1	28.0	69.9
November 9	1.9	19.7	78.4
November 16	3.3	25.1	71.6
November 23	3.0	20.2	76.8
November 30	3.8	27.3	68.9
December 7	6.2	22.3	71.5
December 14	4.1	24.5	71.4
December 21	1.5	18.5	80.0
December 28	1.4	18.4	80.2

For the week of July 13, 2020, 18.7% of all COVID-19 cases (cumulative proportion since the beginning of the pandemic) in the 2 counties were Marshallese, and 45.8% were Latino. By the week of December 28, 2020, the cumulative proportions of all COVID-19 cases since the beginning of the pandemic were reduced to 8.2% among Marshallese and 31.9% among Latino populations.

## Implications for Public Health

The Comprehensive Intervention to Reduce COVID-19 Disparities in Marshallese and Latinx Communities resulted in a substantial decrease in COVID-19 cases among the Marshallese and Latino communities in the 2-county area. Incidence remained lower through the end of December 2020, more closely aligning with the proportions of Marshallese and Latino populations in the 2 counties.

The primary lessons learned from the intervention are the importance of a comprehensive and coordinated approach and the importance of fully engaging the community in communication and outreach to ensure relevance and reach. This intervention was guided by Marshallese and Latino community partners, with coordination and support from academic researchers, local health care professionals, and ADH. By establishing a single coordination center, the intervention ensured that community members were not lost in the transfer among intervention components. Importantly, all communications and intervention materials were provided in English, Marshallese, and Spanish. Furthermore, local community leaders served as the primary spokespeople for health education messages.

Our findings are subject to limitations. These stem from the fact that this public health intervention was rapidly implemented during a pandemic. The intervention was initiated over the course of a few weeks, and we cannot fully account for some confounding factors related to this implementation. First, all incidence of COVID-19 infections presented in this report were limited to laboratory-confirmed cases and excluded all other tests, such as rapid tests, which became increasingly popular at the end of 2020. Second, it was not feasible to control for the potential number of asymptomatic cases or cases that were not confirmed or reported to ADH. Third, some of the observed decline in incidence among the Marshallese population may have been due to continuation of temporal trends that coincided with the implementation of the comprehensive intervention. Fourth, because of the interconnected nature of the multicomponent intervention, it was difficult to identify a specific component of the plan that led to the decrease in cases in each community. Finally, although we do have extensive evidence to document that the Marshallese population has a high prevalence of chronic conditions including obesity and type 2 diabetes, the available data did not allow us to document the proportion of residents with a chronic condition who contracted or died from COVID-19.

Despite these limitations, our findings highlight the importance of building and sustaining CBPR partnerships to address health disparities among groups at high risk for COVID-19. Although our partnerships started because of the high prevalence of type 2 diabetes in the Marshallese and Latino populations, the strength of the partnerships was realized during the COVID-19 pandemic, which was especially devastating for residents with type 2 diabetes. It is rare to see population-level impact of an intervention this quickly and to maintain that impact over several months. Although our findings are unique, the lessons learned are consistent with other studies that demonstrated the importance of CBPR partnerships in rapidly addressing COVID-19 in racial and ethnic minority communities (15,16).
